# The identification of liver metastasis- and prognosis-associated genes in pancreatic ductal adenocarcinoma

**DOI:** 10.1186/s12885-022-09577-2

**Published:** 2022-04-27

**Authors:** Hong Luan, Ye He, Tuo Zhang, Yanna Su, Liping Zhou

**Affiliations:** 1 Department of Laboratory Medicine, The First Affiliated Hospital of China Medical University, Shenyang, Liaoning 110001 People’s Republic of China; 2 Department of Post Graduation Training, The First Affiliated Hospital of China Medical University, No. 155, Nanjingbei Street, Heping District, Shenyang, Liaoning Province 110001 People’s Republic of China

**Keywords:** Liver metastasis, Biomarker, Pancreatic ductal adenocarcinoma, Bioinformatics analysis, SPARC, TPM1

## Abstract

**Background:**

Pancreatic ductal adenocarcinoma (PDAC) is an often fatal malignancy with an extremely low survival rate. Liver metastasis, which causes high mortality, is the most common recurring metastasis for PDAC. However, the mechanisms underlying this liver metastasis and associated candidate biomarkers are unknown.

**Methods:**

We performed mRNA profiling comparisons in 8 primary tumors (T) and 12 liver metastases (M) samples using the Gene Expression Omnibus (GEO) database. After determining differentially expressed genes (DEG), gene ontology (GO), pathway enrichment and protein–protein interaction (PPI) network analyses were performed to determine DEG functions. Then, Cytoscape was used to screen out significant hub genes, after which their clinical relevance was investigated using The Cancer Genome Atlas (TCGA) resources. Furthermore, prognosis-associated gene expression was validated using Oncomine and TCGA database. Lastly, associations between prognosis-associated genes, immune cells and immunological checkpoint genes were evaluated using the Tumor Immune Estimation Resource (TIMER).

**Results:**

In total, 102 genes were related to liver metastasis and predominantly involved in cell migration, motility, and adhesion. Using Cytoscape, this number was narrowed down to 16 hub genes. Elevated mRNA expression levels for two of these genes, SPARC (*P* = 0.019) and TPM1 (*P* = 0.037) were significantly correlated with poor disease prognosis. For the remaining 14, expression was not related to overall patient survival. SPARC had higher expression in patients with metastatic PDAC than those with non-metastatic PDAC in TCGA dataset. SPARC and TPM1 levels were also positively correlated with the immune infiltration of specific cell types. Additionally, both genes exhibited strong co-expression associations with immune checkpoint genes.

**Conclusions:**

Combined, we suggest SPARC has high potential as biomarker to predict liver metastasis during PDAC. Additionally, both SPARC and TPM1 appeared to recruit and regulate immune-infiltrating cells during these pathophysiological processes.

**Supplementary Information:**

The online version contains supplementary material available at 10.1186/s12885-022-09577-2.

## Background

Pancreatic ductal adenocarcinoma (PDAC) is a predominantly fatal malignancy with a very low survival rate of approximately 9% [1]. The online GLOBOCAN (2018) database previously stated pancreatic cancer was the 7^th^ major cause of cancer mortality, with almost as many deaths (*n* = 432,242) as new incidence cases (*n* = 458,918) [2]. A major significant PDAC trait is its extreme aggressiveness, with localized invasion and distant metastasis characteristics. The most frequented distant metastasis site is the liver [3]; in more than 60% of resected patients, relapse was accompanied by distant hepatic recurrence within the first two years post-surgery [4, 5]. Currently, in clinical practice, there are no defined protocols predicting liver metastasis occurrence in individuals with PDAC, therefore, molecular mechanisms must be investigated and potential biomarkers identified for these serious and complex pathophysiological conditions.

The tumor microenvironment (TME) exerts key functions during tumorigenesis, metastasis, and drug resistance towards PDAC [6, 7]. PDAC is non-immunogenic in nature and is characterized by a desmoplastic TME, with high fibroblastic cell numbers and extracellular matrix deposition, poorly infiltrated by CD8^+^ T cells [8]. The structure is highly effective in promoting immune escape, thereby protecting tumor cells against the effective delivery of chemotherapeutic agents [9]. A similar desmoplastic TME also occurs at liver metastatic sites during PDAC [10, 11], however, metastatic TME functions and implications for tumor development and immunotherapy for PDAC are unclear.

Here, we compared mRNA expression profiles between primary tumor and liver metastasis samples using Gene Expression Omnibus (GEO) resources. We performed differentially expressed gene (DEG) analyses using Kyoto Encyclopedia of Genes and Genomes (KEGG) pathway enrichment, protein–protein interaction (PPI) network analyses, and gene ontology (GO) processing to determine gene functions. Then, using the Cytoscape cytoHubba plugin, we screened for the most significant hub genes. Next, we assessed the clinical relevance of hub genes using TCGA. Lastly, associations between liver metastasis and prognosis-associated genes and immune cell types were examined by the Tumor Immune Estimation Resource (TIMER). Our observations provide new knowledge and further insights on liver metastasis mechanisms during PDAC.

## Methods

### Data collection and processing

GEO database (http://www.ncbi.nlm.nih.gov/geo/) is a global public repository for archiving and freely distributing high-throughput gene expression and genomics data sets, which is created by the National Center for Biotechnology Information (NCBI) [12]. The microarray datasets, GSE19279 [13] and GSE42952 [14] from GEO database were used for our studies. GSE19279 comprised 4 primary (T) PDAC and 5 pancreatic liver metastasis (M) samples, whereas GSE42952 included 4 primary (T) PDAC and 7 liver metastasis (M) samples. GEO2R website (http://www.ncbi.nlm.nih. gov/geo/geo2r/) was an analysis tool that comes with the GEO database and was used to screen for differentially expressed mRNAs in M versus T. For analyses, *P* adj. < 0.05 and |log2FC|> 1 were established as cut-off criteria for DEG identification.

### Functional enrichment analyses

Metascape (http://metascape.org) is an effective and efficient online analysis database for gene annotation, functional enrichment, interactome analysis, and membership search [15]. In this study, pathway enrichment analyses (GO, KEGG, BioCarta, and Reactome) of DEGs were conducted using Metascape. The R packages ‘GOplot’ [16], ‘DOSE’ [17], ‘enrichplot’ [18] and ‘clusterProfiler’ [19] (The R Foundation for Statistical Computing, Vienna, Austria) were utilized to implement visualized figures of GO and KEGG enrichment analyses. GO functional enrichment analysis is the major method to explore the potential biological process (BP), molecular function (MF), and cellular component (CC) of genes. KEGG is an important public pathway related biological systems database that integrates genomic, chemical and systemic functional information [20–22]. KEGG pathway enrichment analysis is a common method to identify significantly enriched metabolic pathways or signal transduction pathways in the functional genes. Also, GeneMANIA (http://www.genemania.org/) is an online prediction website for generating hypotheses about gene function, analyzing gene lists and prioritizing genes for functional assays [23]. In the present study, GeneMANIA was used to provide gene networks and co-expressed genes. *P* < 0.05 was established as cut-off criterion.

### Building PPI networks and module analyses

STRING (https://string-db.org/), an online platform of predicted protein interactions, aims to collect, score, and integrate all publicly available sources of PPI information, and to complement these with computational predictions [24]. We conducted a PPI network diagram of identified DEGs with STRING. Those with a combined score ≥ 0.4 were eligible to build the relational network, visualized in Cytoscape (version 3.7.2). Then cytoHubba is a plugin of Cytoscape to rank nodes by their network features in a network, and is utilized to provide 11 analysis methods including DEGREE, Density of Maximum Neighborhood Component (DMNC), Maximal Clique Centrality (MCC), Maximum Neighborhood Component (MNC), Edge Percolated Component (EPC), and six centralities (Bottleneck, Ec-Centricity, Closeness, Radiality, Betweenness, and Stress) [25]. Hub genes were ascertained using five models (DEGREE, DMNC, MCC, MNC, and EPC) in cytoHubba.

### Prognostic hub gene signatures using the TCGA database

TCGA ( https://portal.gdc.cancer.gov/) is a vast repository of high-throughput data on DNA, RNA, and proteins data in many cancer types and multi-omics data that is supported by the National Cancer Institute of the United States [26]. The mRNA expression profile and clinical information of a total of 178 PDAC samples were obtained from the TCGA website for The Cancer Genome Atlas pancreatic ductal adenocarcinoma (TCGA-PAAD) dataset project.

The R ‘survival’ package [27] was used to investigate overall survival analysis. 177 patients with pancreatic cancer were assigned to high (*n* = 88) and low expression groups (*n* = 89) and correlations examined between expression levels and patient survival. One patient had no survival time, excluding survival analysis. Patient prognosis in groups were processed using Kaplan–Meier methods, and survival outcomes in groups were compared using log-rank tests. A log-rank *P* value and a hazard ratio (HR) were calculated and *P* < 0.05 was considered statistically significant.

### Validation of liver metastasis-associated gene expression

Oncomine (https://www.oncomine.org/resource/login.html) is a cancer microarray database and a bioinformatics analysis tool across 18,000 cancer gene expression microarrays which aims at collecting, standardizing, analyzing, and delivering cancer transcriptome data to the biomedical research community [28]. Badea Pancreaes dataset is one of the pancreatic cancer datasets in the Oncomine database. we adopted the Badea Pancreaes dataset to further validate the expression of liver metastasis-associated gene in PDAC.

Gene Expression Profiling Interactive Analysis (GEPIA) (http://gepia.cancer-pku.cn/index.html) is an interactive web server for analyzing the RNA sequencing expression data of 9,736 tumors and 8,587 normal samples from the TCGA and the Genotype-Tissue Expression (GTEx) projects, which is developed by Peking University [29].We also investigated liver metastasis-associated gene expression using GEPIA. Due to limited normal pancreatic tissue at TCGA, we validated specific DEG levels using TCGA PDAC tumor information and matched normal tissue information from TCGA and GTEx platforms. |log2FC|> 1 and *P* < 0.05 values were statistically significant.

UALCAN (http://ualcan.path.uab.edu/analysis.html) is a comprehensive and interactive web resource for analyzing TCGA gene expression data. It allows researchers to study gene expression levels, not only to compare tumor with normal samples, but also to compare across various tumor subgroups based on pathological cancer stages, tumor grade, race, gender, body weight, and other clinicopathologic features [30]. In our study, the UALCAN website was used to determine which clinicopathological factors were related to the expression of liver metastasis-associated gene in PDAC.

One hundred seventy-eighth samples of TCGA-PAAD dataset were divided into non-metastasis and metastasis group, and liver metastasis-associated gene expression levels between the two groups were compared using GraphPad Prism 8.0.2 (GraphPad Prism Software Inc., San Diego, CA, USA). The Mann–Whitney U test was applied to compare the differences between two groups. The data were summarized as median with inter quartile range (IQR). Statistical significance was assumed for a two-tailed *P*-value of < 0.05.

### Connections between liver metastasis-associated genes, immune cell infiltration and immune checkpoints

TIMER (https://cistrome.shinyapps.io/timer/) is a computational tool to comprehensively explore the molecular characterization of tumor immune interactions across diverse cancer [31]. It calculates the abundances of six immune infiltrates (CD8 + T cells, CD4 + T cells, B cells, neutrophils, macrophages, and dendritic cells) based on RNA-Seq expression profiles data. We used TIMER to assess correlations between metastasis-associated gene expression and six immune cells and CTLA4, CD274, PDCD1, and PDCD1LG2 checkpoint genes.

## Results

### DEG screening

**Fig. 1:**
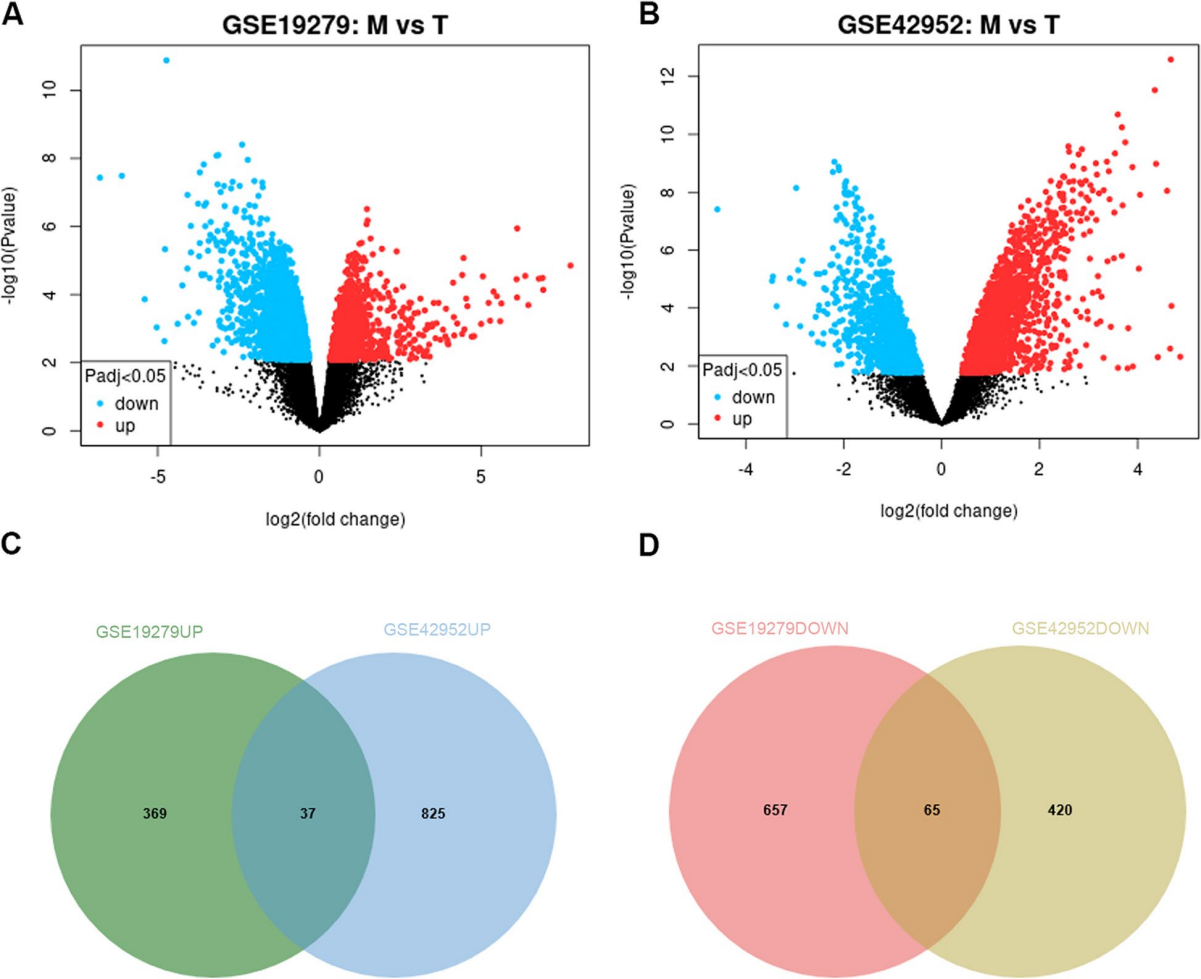
DEGs associated with liver metastasis in PDAC. **A**– **B** Volcano plots were drawn to show the DEGs in primary PDAC (T) samples versus pancreatic liver metastasis (M) samples from the two GEO datasets (GSE19279: 4 primary T vs. 5 M samples, GSE42952: 4 primary T vs. 7 M samples). Red points: upregulated expressed mRNAs; green points: downregulated expressed mRNAs; black points: normally expressed mRNAs. GEO2R online tool was used to analyze DEGs, with the cut‑off criteria of |log2FC|> 1 and adj. *P* < 0.05. Venn diagram displaying **C** 37 upregulated DEGs and **D** 65 downregulated DEGs based on the two GEO datasets

The details of the GEO datasets in this study are shown in supplementary Table S1, and the clinicopathological characteristics of the patients are displayed in Table S2. GEO2R was used to investigate gene expression profiles from GSE19279 and GSE42952 datasets. To identify liver metastasis-associated genes in PDAC, mRNA expression levels were compared in M versus T samples. For analyses, |log2FC|> 1 and *P* < 0.05 were established as statistically meaningful cut-off points. In total, 1128 DEGs, comprising 406 upregulated and 722 downregulated genes were identified in GSE19279, and 1347 DEGs comprising 862 upregulated and 485 downregulated genes were ascertained in GSE42952 (Figs. 1a-b). Venn diagrams showed that 102 genes (37 upregulated and 65 downregulated) overlapped between datasets (Figs. 1c-d), suggesting potentially relevant functions in PDAC metastasis.

### Functional DEG analysis

**Fig. 2:**
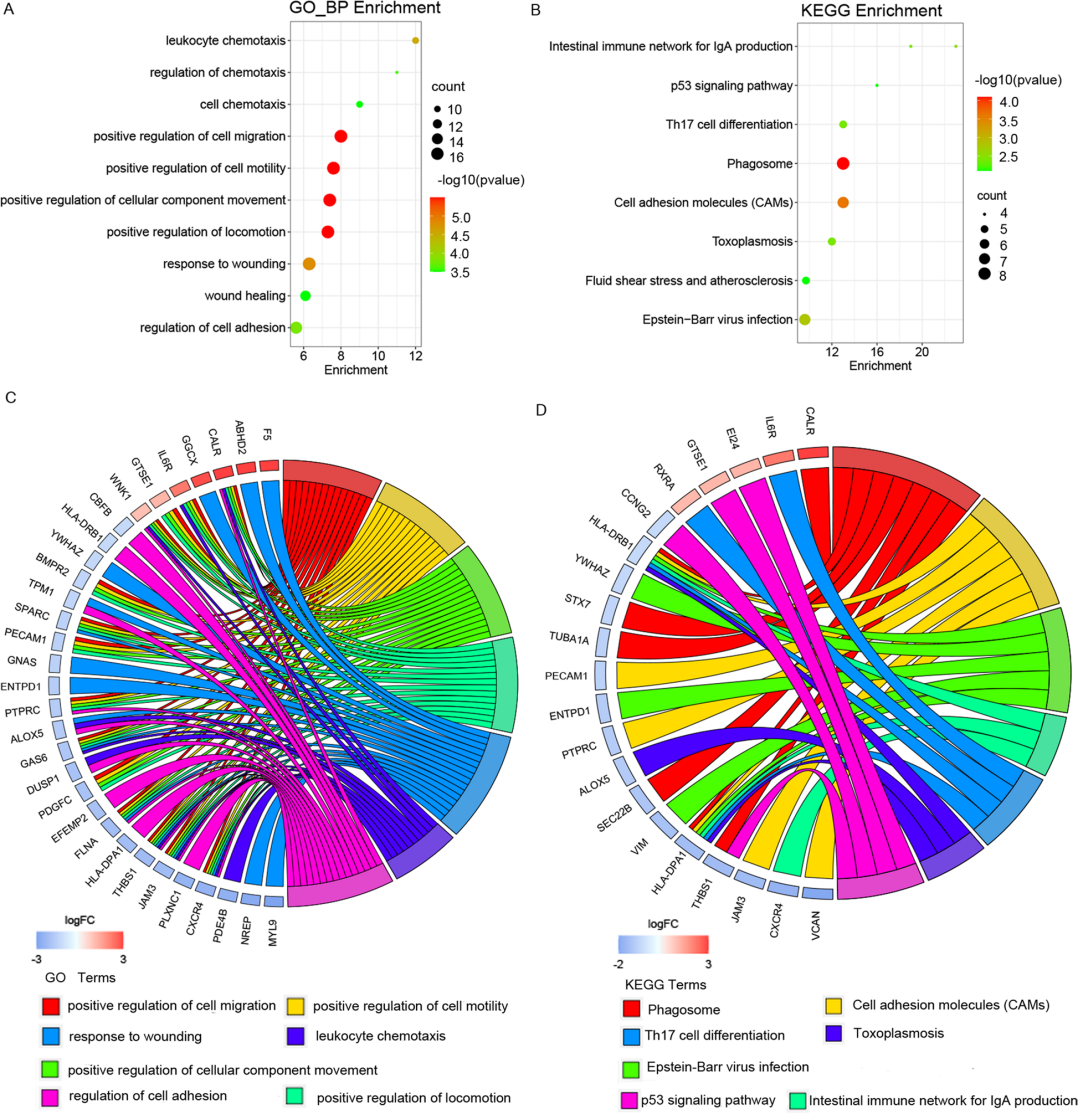
DEGs functional enrichment analysis. GO analysis and KEGG pathway analysis of 102 DEGs were conducted using Metascape database. The R packages were utilized to implement visualized figures of GO and KEGG enrichment analyses. **A** Top 10 enriched GO biological processes for DEGs. **B** Top 10 enriched KEGG pathways for DEGs [20–22]. **C**–**D** Hierarchical clustering of gene expression profiles in GO and KEGG pathway

**Fig. 3:**
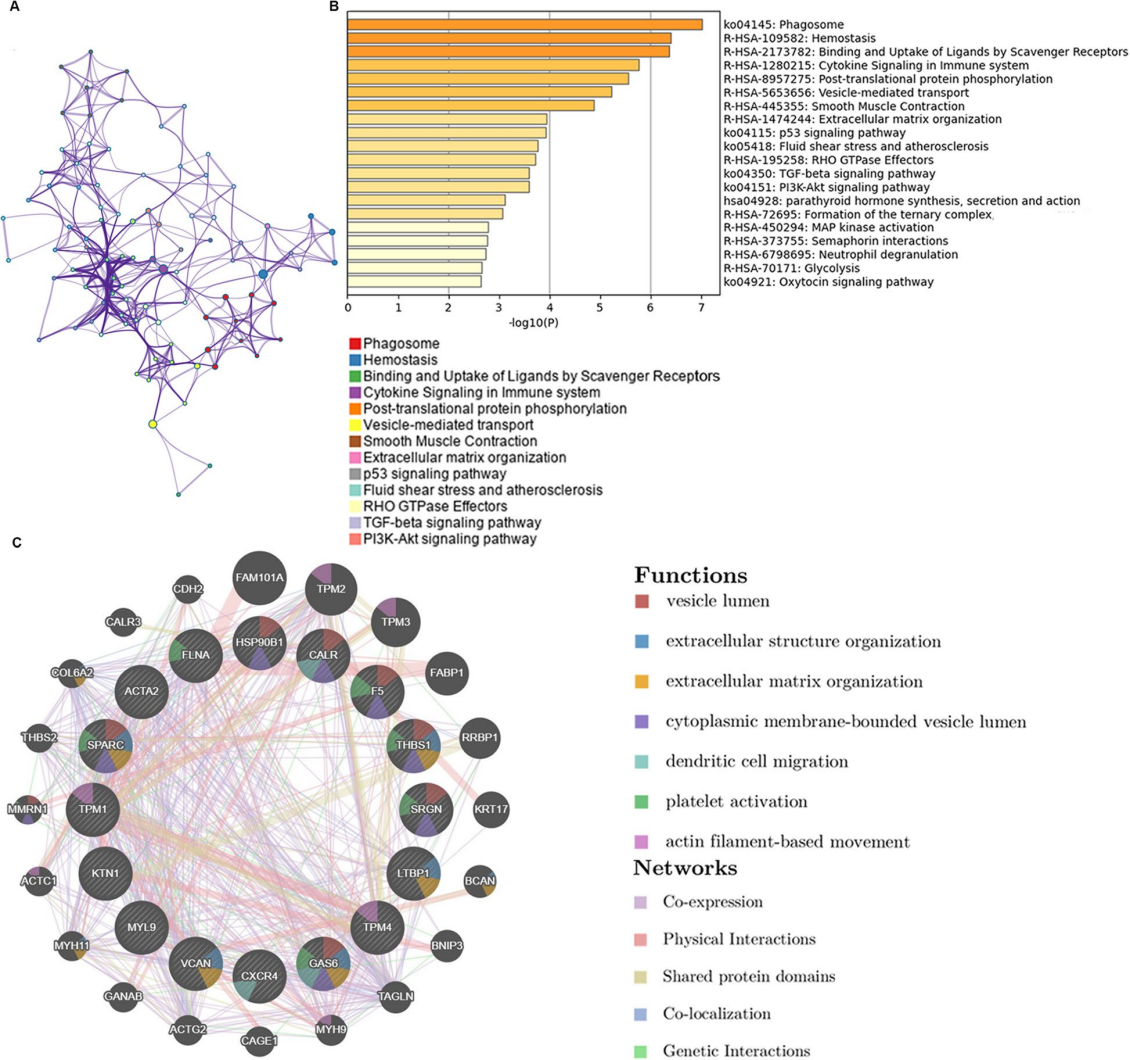
Analysis of hub gene pathways and interaction networks. **A**–**B** Top 20 enriched pathways involving 102 DEGs using Metascape according to KEGG, BioCarta and Reactome. **C** Hub genes and co-expression genes were analyzed using GeneMANIA, 16 hub genes were indicated in the inner circle while 20 of the predicted co-expressed genes were located in the outer circle. The color of the line represented different type of their relationships. The color inside the gene dots illustrated functions which these genes were involved in

From GO investigations, 102 DEGs were primarily positively enriched for the regulation of cell migration, cell motility, cellular component movement, and locomotion (Fig. 2a). KEGG analyses showed DEGs were primarily enriched for phagosome, cell adhesion molecules (CAMs), and Epstein-Barr virus infection (Fig. 2b). GO and KEGG enriched genes are shown in Fig. 2c and d. Pathway analyses using Metascape indicated hub genes were primarily enriched for hemostasis, binding and uptake of ligands by scavenger receptors, cytokine signaling in immune system, and post-translational protein phosphorylation (Figs. 3a-b).

### Building PPI networks and selecting and analyzing hub genes

**Fig. 4:**
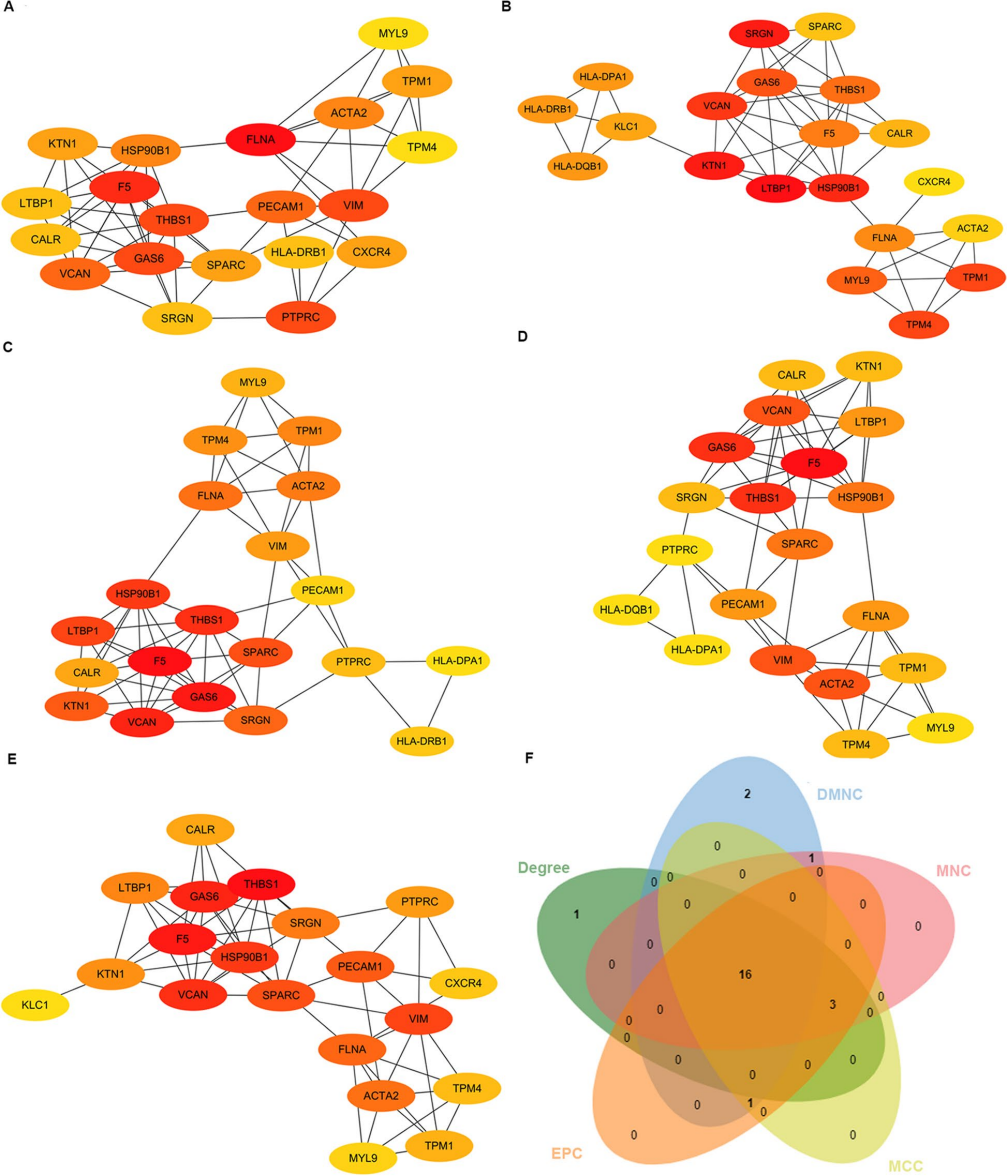
Hub genes identification. **A**–**E** The top 20 hub genes were ascertained using five models (DEGREE, DMNC, MNC, MCC, and EPC) with the Cytoscape (version 3.7.2) plugin cytoHubba based on their connectivity degree. Red represented high degree value and yellow represented low degree value. **F** A Venn diagram showed an overlap of 16 genes

To examine putative DEG interactions, we investigated associations between identified 102 DEGs using STRING. We generated a PPI network of DEGs with combined scores ≥ 0.4. Interacting genes were visualized in Cytoscape for network analysis. Sixteen hub genes were identified based on DEGREE (Fig. 4a), DMNC (Fig. 4b), MCC (Fig. 4c), MNC (Fig. 4d), and EPC (Fig. 4e) Cytoscape models: FLNA, F5, THBS1, GAS6, VCAN, ACTA2, HSP90B1, CXCR4, SPARC, KTN1, TPM1, SRGN, LTBP1, CALR, MYL9 and TPM4 (Fig. 4f). We then used GeneMANIA to analyze these hub genes and their co-expressed genes. All sixteen genes displayed complex PPI networks: 63.82% were co-expression, 24.68% physical interactions, 7.25% shared protein domains, 4.10% were co-localization and 0.16% were Genetic Interactions (Fig. 3c).

### The prognostic relevance of hub genes for PDAC

**Fig. 5:**
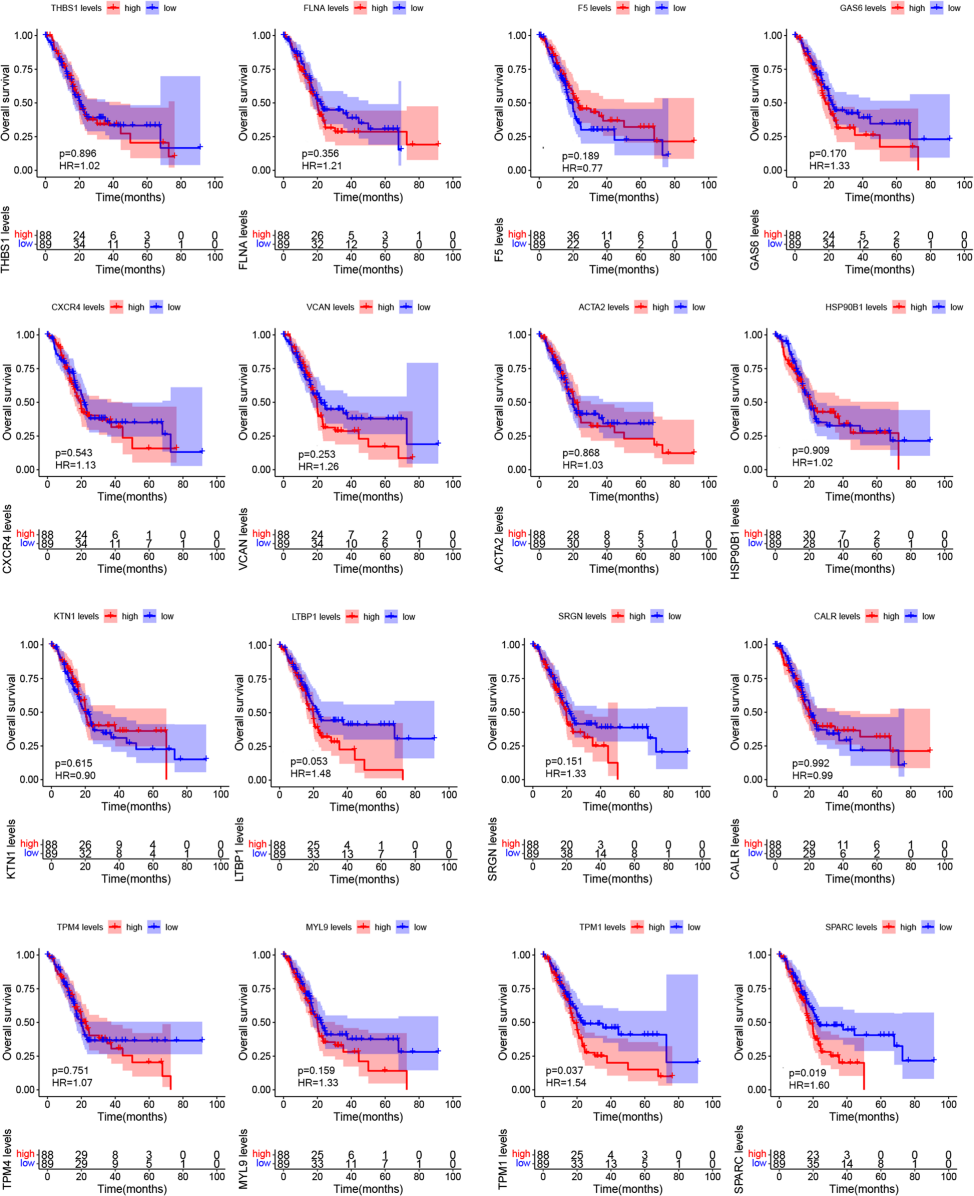
Hub gene expression associations with PDAC prognosis based on the TCGA PAAD datasets (*n* = 177). OS curves of 16 hub genes in TCGA PAAD patients obtained using the Kaplan–Meier method. The patients classified into high- (*n* = 88) or low-expression (*n* = 89) groups for these 16 genes, respectively, and the two groups compared with log-rank test. *P* < 0.05 was considered statistically significant

To evaluate hub gene expression for PDAC prognosis, the TCGA PAAD dataset was used to identify genes associated with overall survival (OS). As indicated (Fig. 5), elevated mRNA expression levels of SPARC (*P* = 0.019, OS HR = 1.60) and TPM1 (*P* = 0.037, OS HR = 1.54) were significantly correlated with poor prognosis. Expression of the remaining 14 genes was not associated with overall patient survival.

### Validation of liver metastasis-associated gene expression using Oncomine, GEPIA and UALCAN database

**Fig. 6:**
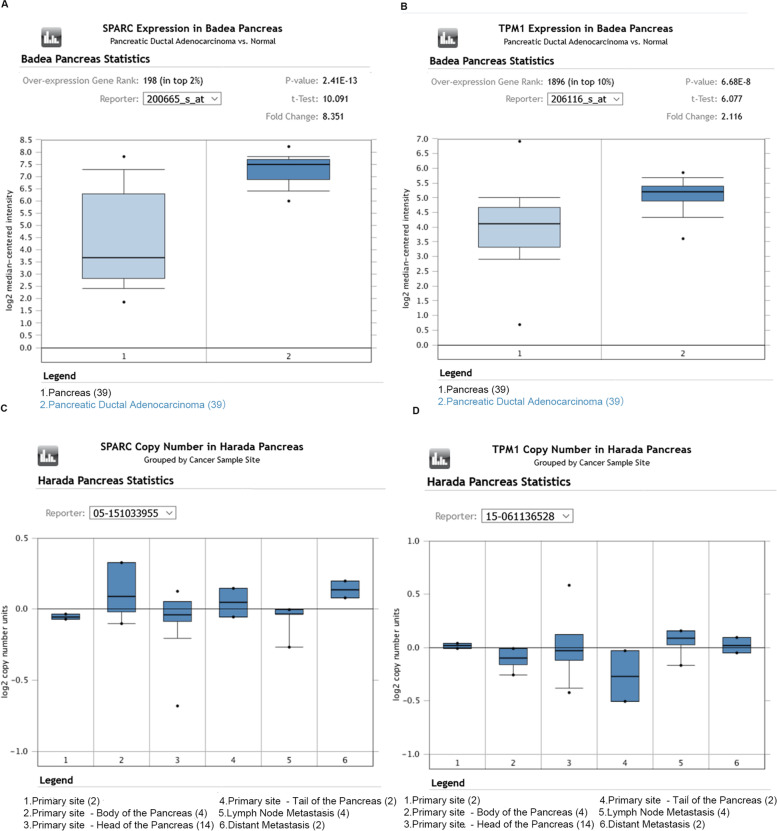
Expression boxplots for SPARC and TPM1 in Oncomine database. **A**-**B** SPARC and TPM1 expression in Badea pancreas (*n* = 78) grouped by normal pancreas (1: *n* = 39) and PDAC (2: *n* = 39). **C**-**D** The expression of SPARC and TPM1 in Harada pancreas (*n* = 28) grouped by cancer sample site (1: primary site, *n* = 2; 2: primary site-body of the pancreas, *n* = 4; 3: primary site-head of the pancreas, *n* = 14; 4: primary site-tail of the pancreas, *n* = 2; 5: lymph node metastasis, *n* = 4; 6: distant metastasis-liver, *n* = 2)

SPARC and TPM1 mRNA expression levels were next verified in PDAC using Oncomine, GEPIA and UALCAN. The Oncomine database revealed that SPARC transcripts were 8.351-fold higher in PDAC when compared with normal samples from Badea Pancreaes Statistics (*P* = 2.41E-13) (Fig. 6a) and TPM1 transcripts were 2.116-fold higher in PDAC comparison to normal samples (*P* = 6.68E-8) (Fig. 6b). Furthermore, SPARC and TPM1 expression were both higher in PDAC liver metastasis tissue than PDAC tissue, however, due to low specimen numbers, *P* values were not calculated (Fig. 6c-d).

GEPIA data also indicated SPARC and TPM1 mRNA levels were significantly elevated in PDAC in comparison to normal pancreatic tissue (Fig. S1).

The correlation of SPARC and TPM1 expression and clinicopathological parameters, including tumor stage, tumor grade and lymphnode metastases, was analyzed by UALCAN database. The results indicated that the expression level of SPARC was higher in grade 2 (*P* = 0.004) and grade 3 (*P* = 0.046) than that in grade 1; the expression level of TPM1 was higher in grade 3 (*P* = 0.043) than that in grade 4 (Fig. S2, Table S3).

To further validate our results, we compared the expression of SPARC and TPM1 between metastasis and non-metastasis PDAC patients in TCGA dataset. The results showed that SPARC had higher expression in the metastasis group than that in the non-metastasis group. (*P* = 0.041) (Fig. S3).

### SPARC and TPM1 relevance to immune cell infiltration in PDAC

**Fig. 7:**
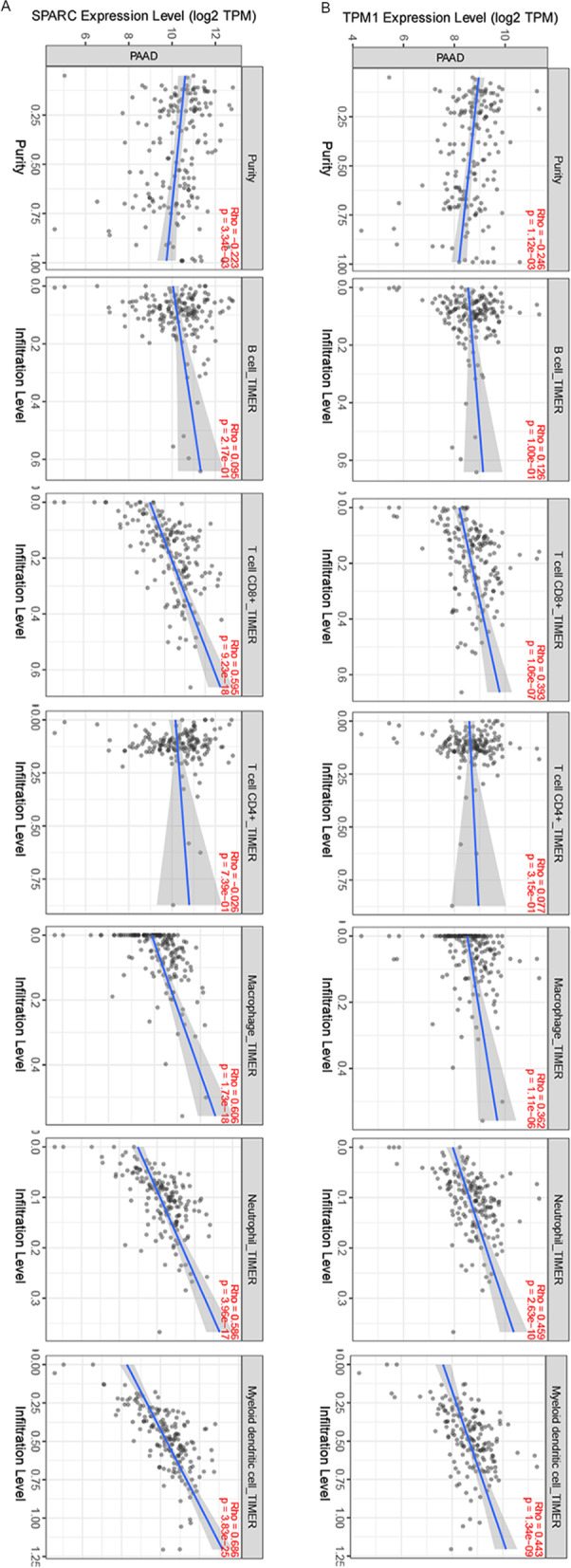
Analysis of immune cell infiltration. SPARC (**A**) and TPM1 (**B**) expression correlations with tumor purity and infiltrating levels of B cells, CD8 + T cells, CD4 + T cells, macrophages, neutrophils, and dendritic cells in TIMER database. The scatter plots displayed the purity-adjusted spearman’s rho value and statistical significance

**Fig. 8:**
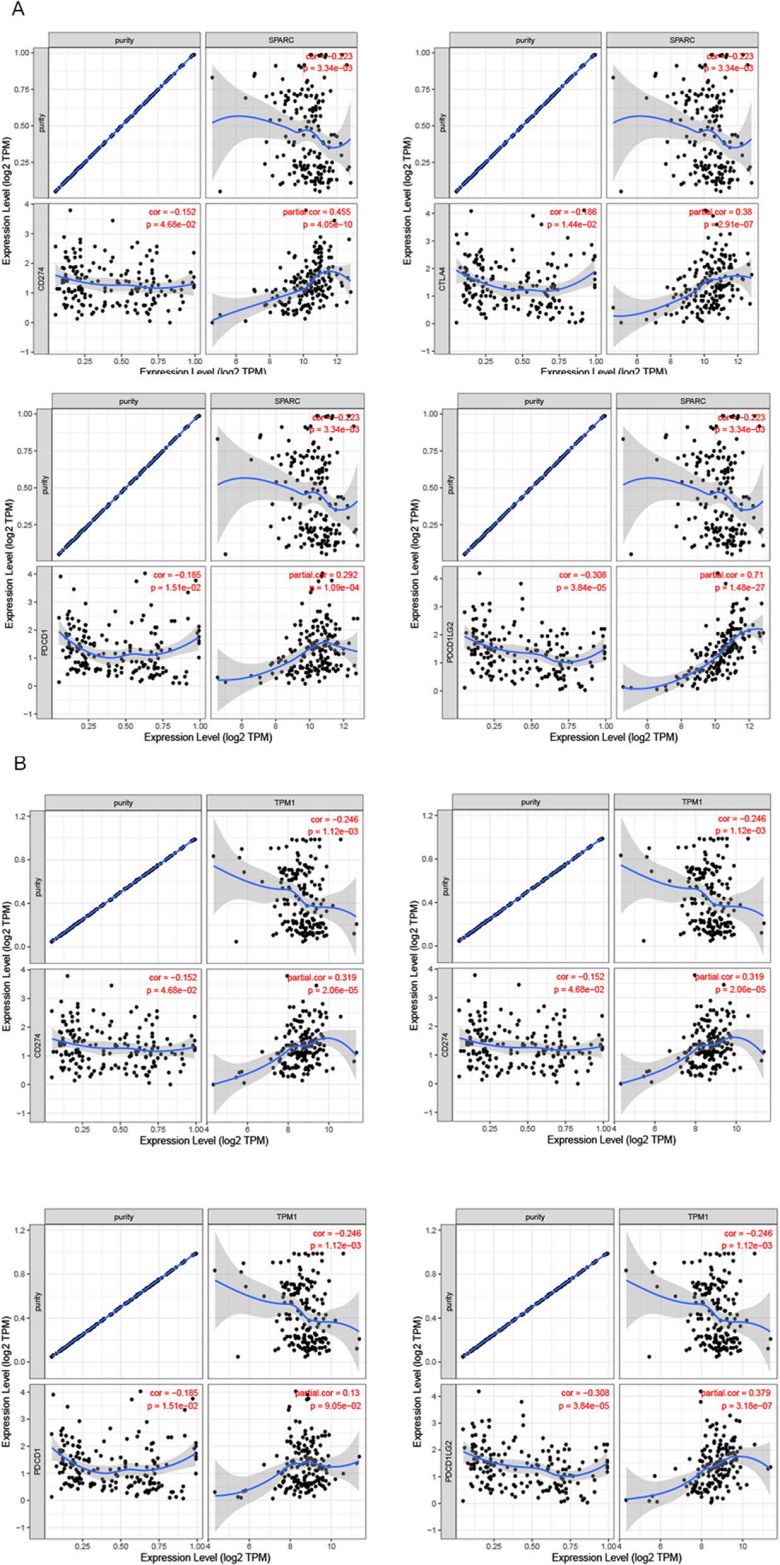
Association between SPARC (**A**) and TPM1 (**B**) expression level and immune checkpoints genes (CD274, CTLA4, PDCD1, and PDCD1LG2) using TIMER (*n* = 368)

To ascertain if correlations existed between tumor infiltrating immune cells and metastasis-associated gene levels, we examined associations using TIMER. SPARC expression was positively correlated with infiltrating CD8 + T cells (*P* = 9.23e-18), macrophages (*P* = 1.73e-18), neutrophil (*P* = 3.96e-17), and myeloid dendritic cells (*P* = 3.83e-25). Similarly, TPM1 was also positively correlated with infiltrating CD8 + T cells (*P* = 1.06e-07), macrophages (*P* = 1.11e-06), neutrophil (*P* = 2.63e-10), and myeloid dendritic cells (*P* = 1.34e-09) (Fig. 7). As current immunotherapy strategies rely on immunological checkpoint inhibitors [32, 33], we used TIMER to investigate co-expression relationship of both genes with immune checkpoint-related genes. SPARC displayed strong co-expression relationships with CD274, CTLA4, PDCD1, and PDCD1LG2, whereas TPM1 had co-expression relationships with CD274, CTLA4, and PDCD1LG2 (Fig. 8).

## Discussion

Liver metastasis is a critical issue during PDAC and adversely affects patient survival and prognosis. Therefore, metastasis mechanisms must be unraveled and new candidate biomarkers identified to comprehensively monitor this harmful pathophysiology.

Here, mRNA expression levels in M and T tissue were compared and showed that 102 DEGs were specifically associated with liver metastasis. Our GO enrichment analyses indicated that DEGs were implicated in regulating cell migration, motility, cell component movement, and locomotion. Our KEGG analyses also determined that DEGs were mostly enriched for the phagosome, CAMs, and Epstein-Barr virus infection. Cell migration, including single cell and collective cell models, is a key step in mediating carcinoma cell metastatic dissemination [34]. CAMs through multifaceted roles as signaling molecules, mechanotransducers and key components of the cell migration machinery are involved in almost every step of cancer progression from primary tumor development to metastasis[35, 36]. Based on these studies, we hypothesize DEGs may promote liver metastasis during PDAC by regulating cell adhesion, movement, and invasion processes.

Next, we examined the prognostic relevance of the identified 16 hub genes. These data suggested elevated SPARC and TPM1 expression levels were highly associated with a poor PDAC prognosis, indicating their putative value as prognostic PDAC indicators. Since the overview of Table S2 shows "NA" for most of pathological data, we could not analyze the correlation of survival and prognosis with the clinical data of patients in this manuscript. Subsequently, clinical samples are needed to be collected to verify the correlation between prognosis and clinical parameters.

The immune system, particularly participating tumor infiltrating immune cells, is involved in tumor metastasis [6, 37], therefore we predicted gene-immune cell interactions with identified hub genes. Both SPARC and TPM1 expression levels were significantly associated with infiltrating CD8 + T cells, macrophages, neutrophils, and dendritic cells. Also, both genes demonstrated strong co-expression relationships with immunological checkpoint genes. Combined, SPARC and TPM1 may be involved in recruiting and regulating immune-infiltrating cells during liver metastasis in PDAC. However, SPARC and TPM1 roles in tumor-infiltrating require further investigation.

Functioning as an extracellular protein, SPARC has essential functions in cancer cell proliferation, migration, angiogenesis, matrix cell adhesion, and tissue remodeling [38, 39]. The protein is found in tumor stroma and is elevated in several cancers, including breast [40], lung [41, 42], and melanoma [43]. Furthermore, elevated SPARC expression at the stoma is present in approximately 40% of patients with PDAC having received resection surgery, and critically, SPARC levels appear to be an independent prognostic factor [44]. A previous study also indicated that polymorphisms in SPARC could predict outcomes for patients with locally advanced and metastatic pancreatic cancer [45]. In another study, SPARC had a dual functional role as a prognosis predictor and potential marker for lymph node metastasis in patients with resected lesions [46]. In our study, SPARC was found to be have higher expression in the metastasis group than that in the non-metastasis group in PDAC patients, and high expression indicated shorter OS. This suggested that SPARC could be use as biomarker of PDAC liver metastasis. However, precise SPARC functions in PDAC related liver metastasis remain unclear.

As a member of the tropomyosin (TM) family, TPM1 encodes high molecular weight TM isoforms which regulate the proliferation, motility, invasion, and metastasis of tumor cells [47]. Studies have shown TPM1 expression is dysregulated across several carcinomas, including gastric [48], bladder [49] and osteosarcoma[50]. Low TPM1 expression predicted reduced survival in gastric cancer[48]. Tang et al*.* reported that TPM1 was elevated in renal cell carcinoma cell line, where tumor cell apoptosis was induced via p53-mediated mitochondrial signaling [51]. In this study, we found that elevated TPM1 expression was associated with poor prognosis in PDAC patients and TPM1 involved in recruiting and regulating immune-infiltrating cells during PDAC metastasis, but the expression of TPM1 was not statistically significant between metastatic and non-metastatic PDAC patients. TPM1 seems to not be a biomarker of PDAC live metastasis. However, TPM1 functions in pancreatic cancer and correlations with PDAC mediated liver metastasis need further study.

While our study still has some limitations, first, due to the lack of the clinicopathological parameters of patients in the GEO database, we could not collect enough data for effective analysis. Second, only the analysis of bioinformatics, we believe more cytological experiments, animal experiments and clinical studies must be conducted to experimentally verify our observations.

## Conclusions

Using an integrated bioinformatics analysis approach, sixteen hub genes associated with liver metastasis PDAC were identified and their functions and pathways investigated. Our data suggested the two hub genes, SPARC and TPM1 may have key roles in the PDCA tumor microenvironment by regulating tumor-infiltrating immune cells. Further studies will be required to comprehensively explore SPARC and TPM1 functions and mechanisms in PDAC liver metastatic processes.

## Supplementary Information

**Additional file 1:** **Figure S1.** SPARC and TPM1 expression analysis in PADC based on GEPIA. **Figure S2.** The correlation of SPARC and TPM1 expression and clinicopathological parameters using the UALCAN database. **Figure S3.** Compared the expression of SPARC and TPM1 between metastasis and non-metastasis PDAC patients in TCGA dataset. **Table S1.** Information on the Microarray datasets. **Table S2.** Clinicopathological characteristics of the patients with PDAC. **Table S3.** The corrections of SPARC and TPM1 and tumor stage, lymphnode metastases and tumor grade. 

## Data Availability

The data used to support the findings of this study were available from the Gene Expression Omnibus (http://www.ncbi.nlm.nih.gov/geo) and Cancer Genome Atlas database (https://cancergenome.nih.gov).

## Abbreviations

PDAC: Pancreatic ductal adenocarcinoma
TME: Tumor microenvironment
GEO: Gene Expression Omnibus
DEG: Differentially expressed gene analyses
KEGG: Kyoto Encyclopedia of Genes and Genomes
PPI: Protein–protein interaction
GO: Gene ontology
TIMER: Tumor Immune Estimation Resource
HR: Hazard ratio
GEPIA: Gene Expression Profiling Interactive Analysis
GTEx: Genotype-Tissue Expression
TCGA-PAAD: The Cancer Genome Atlas pancreatic ductal adenocarcinoma
IQR: Inter quartile range
CAMs: Cell adhesion molecules
TM: Tropomyosin
OS: Overall survival
