# Supplementary material for: The identification of liver metastasis- and prognosis-associated genes in pancreatic ductal adenocarcinoma
Source: BMC Cancer. 2022 Apr 27;22:463. doi: 10.1186/s12885-022-09577-2 (PMC9047343; doi:10.1186/s12885-022-09577-2)
Supplement: Supplementary file 1 — Additional file 1: Figure S1. SPARC and TPM1 expression analysis in PADC based on GEPIA. Figure S2. The correlation of SPARC and TPM1 expression and clinicopathological parameters using the UALCAN database. Figure S3. Compared the expression of SPARC and TPM1 between metastasis and non-metastasis PDAC patients in TCGA dataset. Table S1. Information on the Microarray datasets. Table S2. Clinicopathological characteristics of the patients with PDAC. Table S3. The corrections of SPARC and TPM1 and tumor stage, lymphnode metastases and tumor grade. [file 12885_2022_9577_MOESM1_ESM.docx]

**Supplementary Materials**

Figure S1: SPARC and TPM1 expression analysis in PADC based on GEPIA.

Figure S2: The correlation of SPARC and TPM1 expression and clinicopathological parameters using the UALCAN database.

Figure S3: Compared the expression of SPARC and TPM1 between metastasis and non-metastasis PDAC patients in TCGA dataset.

Table S1: Information on the Microarray datasets.

Table S2: Clinicopathological characteristics of the patients with PDAC.

Table S3: The corrections of SPARC and TPM1 and tumor stage, lymphnode metastases and tumor grade.

**Supplementary figures**


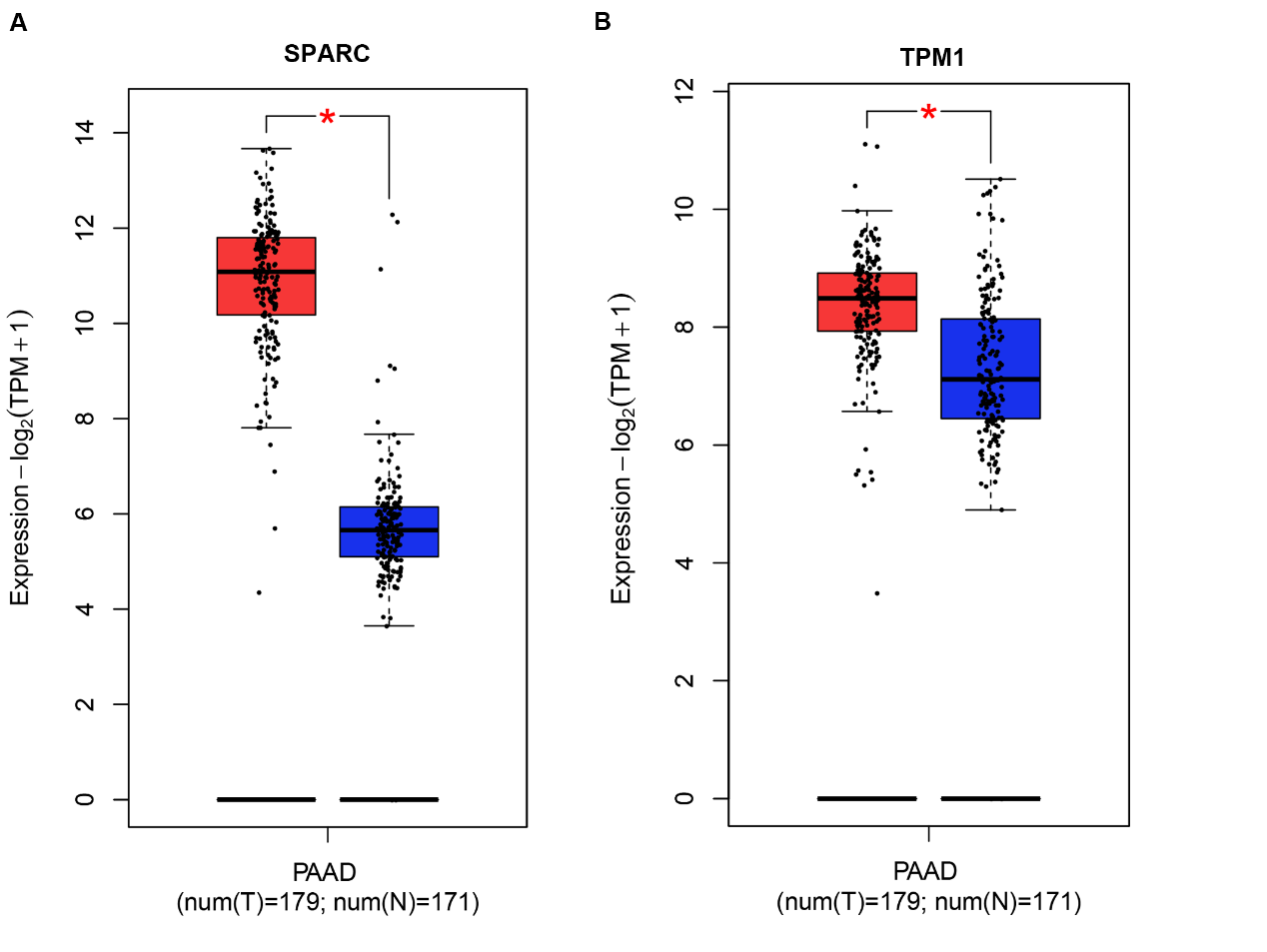


**Figure S1.** SPARC and TPM1 expression analysis in PADC based on GEPIA. SPARC mRNA (A) and TPM1 mRNA expression (B) in TCGA pancreatic cancer tumor (n = 179) and matching normal tissue (n = 171) from data of TCGA and GTEx. A *P* value < 0.05 was statistically significant.


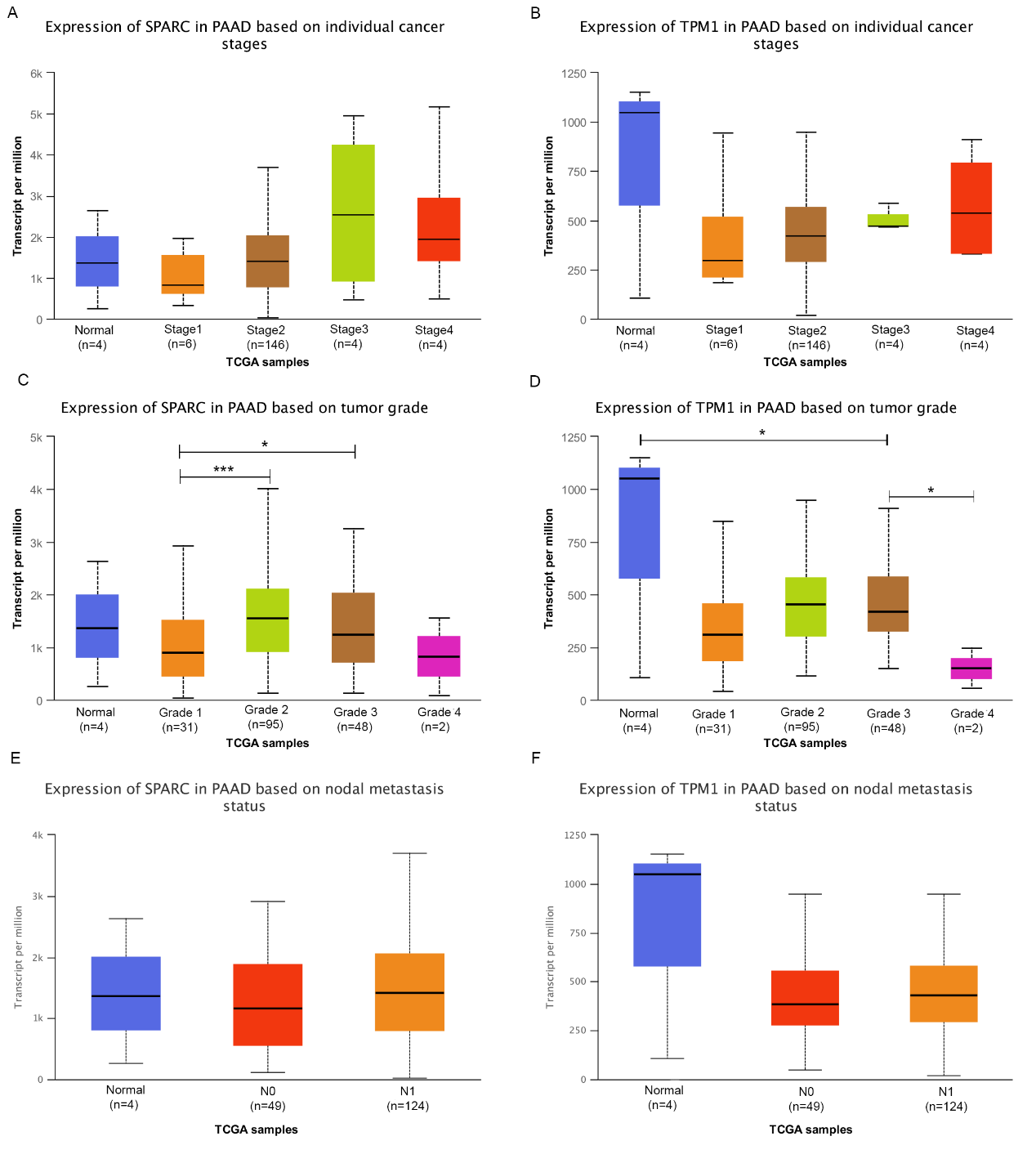


**Figure S2.** The correlation of SPARC and TPM1 expression and clinicopathological parameters using the UALCAN database. (A-B) Expression of SPARC and TPM1 in PAAD based on individual cancer stages (n = 164). (C-D) Expression of SPARC and TPM1 in PAAD based on tumor grade (n = 180). (E-F) Expression of SPARC and TPM1 in PAAD based on nodal metastasis status (n = 177). ^∗^*P* < 0.05; ^∗∗^*P* < 0.01; ^∗∗∗^*P* < 0.001.


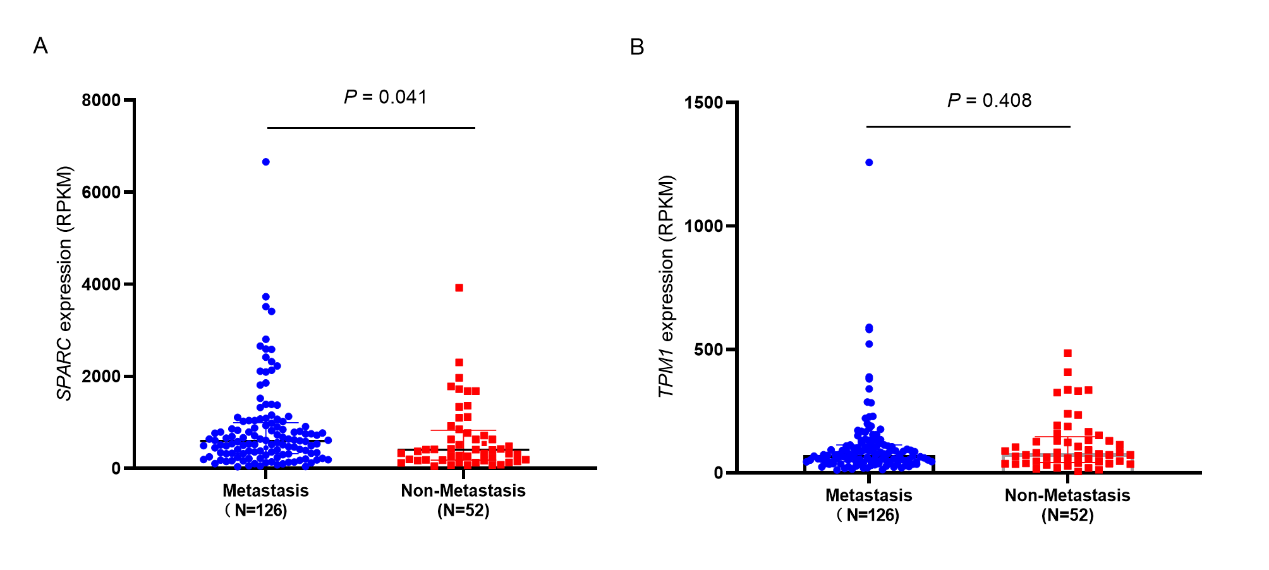


**Figure S3.** Compared the expression of SPARC and TPM1 between metastasis and non-metastasis PDAC patients in TCGA dataset. (A) The expression of SPARC in metastatic (median: 593.73; IQR: 318.61-991.03; n =126) and non-metastatic (median: 403.80; IQR: 182.00‐829.41; n = 52) PDAC samples from TCGA database. (B) The expression of TPM1 in metastatic (median: 73.57; IQR: 45.49‐113.20; n =126) and non-metastatic (median: 79.17; IQR: 41.63‐146.64; n = 52) PDAC samples from TCGA database. *P* < 0*.*05 was considered statistically significant.

**Supplementary Table S1.** Information on the Microarray datasets.

|  |  | GSE19279 | GSE42952 |
| --- | --- | --- | --- |
| Type of microarray |  | Expression profiling by array | Expression profiling by array |
| Total samples |  | 15 | 33 |
|  | original tumor tissue of PDAC(T) | 4 | 4 |
|  | normal pancreatic tissue | 3 | 6 |
|  | normal liver tissue | 3 | 0 |
|  | liver metastasis (LM) | 5 | 7 |
|  | peritoneal metastasis (PM) | 0 | 4 |
|  | others | 0 | 12 |
| Selected samples |  | 9 | 11 |
|  | original tumor tissue of PDAC(T) | 4 | 4 |
|  | liver metastasis (LM) | 5 | 7 |
| Platform |  | GPL96[HG-U133A] Affymetrix Human Genome U133A Array | GPL570[HG-U133_Plus_2] Affymetrix Human Genome U133 Plus 2.0 Array |
| Responsible institutions |  | Barts & The London School of Medicine (QMUL)Institute of Cancer | VIB Genomics Core, University of Leuven (KU Leuven) |
| Submission date |  | Dec 02, 2009 | Dec 17, 2012 |
| Country |  | United Kingdom | Belgium |
| Reference |  | Barry S et al. (2013) [12] | Van den Broeck et al. (2013) [13] |

**Supplementary Table S2.** Clinicopathological characteristics of the patients with PDAC.

| Sample code | Date set | Accession | Age(yr) | Gender | Tumor grade | T-stage | N-stage | M-stage | AJCC | Tissue |
| --- | --- | --- | --- | --- | --- | --- | --- | --- | --- | --- |
| P1 | GSE19279 | GSM478458 | NA | NA | NA | NA | NA | M1 | NA | original tumor tissue of PDAC |
| P2 | GSE19279 | GSM478459 | NA | NA | NA | NA | NA | M1 | NA | original tumor tissue of PDAC |
| P5 | GSE19279 | GSM478460 | NA | NA | NA | NA | NA | M1 | NA | original tumor tissue of PDAC |
| P6 | GSE19279 | GSM478461 | NA | NA | NA | NA | NA | M1 | NA | original tumor tissue of PDAC |
| M1 | GSE19279 | GSM478465 | NA | NA | NA | NA | NA | M1 | NA | liver metastasis from pancreatic cancer |
| M2 | GSE19279 | GSM478466 | NA | NA | NA | NA | NA | M1 | NA | liver metastasis from pancreatic cancer |
| M3 | GSE19279 | GSM478467 | NA | NA | NA | NA | NA | M1 | NA | liver metastasis from pancreatic cancer |
| M4 | GSE19279 | GSM478468 | NA | NA | NA | NA | NA | M1 | NA | liver metastasis from pancreatic cancer |
| M5 | GSE19279 | GSM478469 | NA | NA | NA | NA | NA | M1 | NA | liver metastasis from pancreatic cancer |
| T105 | GSE42952 | GSM463731 | 68 | male | G3 | T2 | N0 | M0 | ⅠB | original tumor tissue of PDAC |
| T91 | GSE42952 | GSM463735 | 51 | female | G3 | T3 | N0 | M0 | ⅡA | original tumor tissue of PDAC |
| T138 | GSE42952 | GSM463739 | 50 | male | G2 | T2 | N1 | M0 | ⅡB | original tumor tissue of PDAC |
| T123 | GSE42952 | GSM463743 | 74 | male | G3 | T1 | N1 | M0 | ⅡB | original tumor tissue of PDAC |
| 2-45 | GSE42952 | GSM1053827 | 69 | male | NA | NA | NA | M1 | NA | liver metastasis from pancreatic cancer |
| 4-M1 | GSE42952 | GSM1053831 | 69 | male | NA | NA | NA | M1 | NA | liver metastasis from pancreatic cancer |
| 6-M2 | GSE42952 | GSM1053835 | 65 | female | NA | NA | NA | M1 | NA | liver metastasis from pancreatic cancer |
| 9-167 | GSE42952 | GSM1053839 | 62 | female | NA | NA | NA | M1 | NA | liver metastasis from pancreatic cancer |
| 11-179 | GSE42952 | GSM1053842 | 69 | male | NA | NA | NA | M1 | NA | liver metastasis from pancreatic cancer |
| 12-180 | GSE42952 | GSM1053844 | 47 | male | NA | NA | NA | M1 | NA | liver metastasis from pancreatic cancer |
| 17-TM2 | GSE42952 | GSM1053847 | 67 | male | NA | NA | NA | M1 | NA | liver metastasis from pancreatic cancer |
| Abbreviations: NA, Not Available | | | | | | | | | | |

**Supplementary Table S3. The corrections of SPARC and TPM1 and tumor stage, lymphnode metastases and tumor grade.**

|  | Tumor stage | | Tumor Grade | | Nodal metastases | |
| --- | --- | --- | --- | --- | --- | --- |
|  | Comparison | Statistical significance | Comparison | Statistical significance | Comparison | Statistical significance |
| SPARC |  |  |  |  |  |  |
|  | Normal-vs-Stage1 | 0.5583 | Normal-vs-Grade 1 | 0.7071 | Normal-vs-N0 | 0.9410 |
|  | Normal-vs-Stage2 | 0.7663 | Normal-vs-Grade 2 | 0.2836 | Normal-vs-N1 | 0.7015 |
|  | Normal-vs-Stage3 | 0.4320 | Normal-vs-Grade 3 | 0.3911 | N0-vs-N1 | 0.2840 |
|  | Normal-vs-Stage4 | 0.4903 | Normal-vs-Grade 4 | 1.0000 |  |  |
|  | Stage1-vs-Stage2 | 0.2487 | Grade 1-vs-Grade 2 | 0.0004 |  |  |
|  | Stage1-vs-Stage3 | 0.2558 | Grade 1-vs-Grade 3 | 0.0463 |  |  |
|  | Stage1-vs-Stage4 | 0.2835 | Grade 1-vs-Grade 4 | 0.7071 |  |  |
|  | Stage2-vs-Stage3 | 0.4303 | Grade 2-vs-Grade 3 | 0.2646 |  |  |
|  | Stage2-vs-Stage4 | 0.2252 | Grade 2-vs-Grade 4 | 0.2836 |  |  |
|  | Stage3-vs-Stage4 | 0.8788 | Grade 3-vs-Grade 4 | 0.3911 |  |  |
| TPM1 |  |  |  |  |  |  |
|  | Normal-vs-Stage1 | 0.2480 | Normal-vs-Grade 1 | 0.4135 | Normal-vs-N0 | 0.4205 |
|  | Normal-vs-Stage2 | 0.1407 | Normal-vs-Grade 2 | 0.1213 | Normal-vs-N1 | 0.1463 |
|  | Normal-vs-Stage3 | 0.2888 | Normal-vs-Grade 3 | 0.0428 | N0-vs-N1 | 0.2768 |
|  | Normal-vs-Stage4 | 0.5917 | Normal-vs-Grade 4 | 1.0000 |  |  |
|  | Stage1-vs-Stage2 | 0.6257 | Grade 1-vs-Grade 2 | 0.2993 |  |  |
|  | Stage1-vs-Stage3 | 0.9627 | Grade 1-vs-Grade 3 | 0.5240 |  |  |
|  | Stage1-vs-Stage4 | 0.4123 | Grade 1-vs-Grade 4 | 0.4135 |  |  |
|  | Stage2-vs-Stage3 | 0.6510 | Grade 2-vs-Grade 3 | 0.4297 |  |  |
|  | Stage2-vs-Stage4 | 0.5528 | Grade 2-vs-Grade 4 | 0.1213 |  |  |
|  | Stage3-vs-Stage4 | 0.3762 | Grade 3-vs-Grade 4 | 0.0428 |  |  |
